# Impact of Apolipoprotein E gene polymorphism during normal and pathological conditions of the brain across the lifespan

**DOI:** 10.18632/aging.101757

**Published:** 2019-01-24

**Authors:** Diego Iacono, Gloria C. Feltis

**Affiliations:** 1 Neuropathology Research, Biomedical Research Institute of New Jersey (BRInj), Cedar Knolls, NJ 07927, USA; 2 MidAtlantic Neonatology Associates (MANA), Morristown, NJ 07960, USA; 3 Atlantic Neuroscience Institute, Atlantic Health System (AHS), Overlook Medical Center, Summit, NJ 07901, USA

**Keywords:** *APOE*-polymorphism, brain development and aging, neural cells ratios, environmental modifiers, in-utero and centenarian life

## Abstract

The central nervous system (CNS) is the cellular substrate for the integration of complex, dynamic, constant, and simultaneous interactions among endogenous and exogenous stimuli across the entire human lifespan. Numerous studies on aging-related brain diseases show that some genes identified as risk factors for some of the most common neurodegenerative diseases - such as the allele 4 of *APOE* gene (*APOE4*) for Alzheimer’s disease (AD) - have a much earlier neuro-anatomical and neuro-physiological impact. The impact of *APOE* polymorphism appears in fact to start as early as youth and early-adult life. Intriguingly, though, those same genes associated with aging-related brain diseases seem to influence different aspects of the brain functioning much earlier actually, that is, even from the neonatal periods and earlier. The *APOE4*, an allele classically associated with later-life neurodegenerative disorders as AD, seems in fact to exert a series of very early effects on phenomena of neuroplasticity and synaptogenesis that begin from the earliest periods of life such as the fetal ones.

We reviewed some of the findings supporting the hypothesis that *APOE* polymorphism is an early modifier of various neurobiological aspects across the entire human lifespan - from the in-utero to the centenarian life - during both normal and pathological conditions of the brain.

## Introduction

Some genes identified as risk factors for aging-related neurodegenerative diseases - such as the allele 4 of Apolipoprotein E gene (*APOE4*) for Alzheimer’s disease (AD) - can have an early impact on different neuronal and non-neuronal features of the human brain, even starting from the perinatal periods or earlier. The possible early influence of *APOE* polymorphism on various cognitive and non-cognitive aspects through the entire human lifespan [1–8] poses the biological rationale for a wider re-consideration on how identical genes - for example, the ones identified as risk factors for aging-related diseases - could differently interact with other genes and environmental factors from the beginning of the human life until the advanced or extreme age.

Essentially, in this context, the main scientific question is: how do these genetic risk factors for late-life brain diseases impact, or have impacted, the general development of the brain and specific cytoarchitectural aspects of neuronal and non-neuronal cells and generate, or have generated, meaningful changes on complex cerebral functions (e.g. episodic memory, visual attention) and ultimately determine divergent clinical outcomes across the entire human lifespan [9,10].

It is in fact more and more evident that a series of mutual and long-term interactions between genetic and environmental factors control, or might even determine, quantitative differences between healthy and pathologic neural tissues [11–13]. Accordingly, it would be only after a relatively long period of time, normally measured in decades, that global or more selective quantitative differences across different types of neural circuits (e.g. extrapyramidal motor system, limbic system, pyramidal motor system) or brain regions (e.g. hippocampus, amygdala, frontal cortex), would affect specific cerebral functions later in life. Specifically, these cellular quantitative differences could culminate later in life in a more diffuse and irreversibly progressive neurodegenerative process such as dementia (e.g. Alzheimer’s disease [AD], dementia with Lewy bodies [DLB], frontotemporal dementias [FTDs]); in some more selectively distributed deficits (e.g. Parkinson’s disease [PD], progressive supranuclear palsy [PSP]); or in a more focally-distributed functional impairment of a specific neuronal circuitry (e.g. amnestic mild cognitive impairment [a-MCI], essential tremor [ET]).

The concept about gene-environment influence on quantitative ratios between health and pathologic neural tissues implies that when certain specific amounts of neuronal and non-neuronal cells (e.g. glial), which we could term “cellular ratio thresholds” that support the global functioning of the brain (e.g. cardio-respiratory regulation through the cardio-respiratory circuits of the brainstem nuclei), or a cluster of specific cerebral functions (e.g. the executive functions through the signals elaboration in the frontal cortex, or the visual-spatial functions through the temporal lobe activation), or a single cerebral function (e.g. the memory through the hippocampal neuronal firing integration) in a specific individual (a subject carrying a specific set of genes and exposed to either beneficial or detrimental environmental factors along his/her previous life) have been crossed, a neurodegenerative process begins to be clinically manifest and, only in appearance, to be linked to the aging process, but actually being the effect of a long-lasting accrual of various pathogenic and detrimental elements accumulated in the previous decades of his/her life.

Maintaining adequate cellular ratios between healthy and pathologic neural tissues would consist in maintaining enough amounts of still-functioning (normal) *vs.* non-functioning (pathologic) cells able to keep and preserve a normal operational status of the brain. This normal operational status would be the one accounting for keeping motor, sensory, cognitive, emotional, and behavioral skills still functional enough in order to conduct the normal daily functions of life in an independent manner [14]. Importantly, these functional neural ratios (neuronal and non-neuronal cells) kept below the “dysfunctional cellular ratio thresholds” would not be simply determined by the passive accumulation of a single or multiple brain pathologies (e.g. accumulation of extracellular insoluble β-amyloid in the cerebral cortex or intracellular formation of hyperphosphorylated-tau neurofibrillary tangles as in AD) but also on how certain “predisposing genes” in a specific CNS/brain/person have mutually interacted with a specific group of environmental factors throughout his/her life.

One of the most striking examples of the possibility to maintain normally-functioning neural ratios despite the presence of brain lesions accumulation, it is observation that some older subjects can be categorized as asymptomatic AD subjects (ASYMAD). ASYMAD are cognitively normal older subjects that show, at autopsy, an equivalent or even higher amounts of AD pathology in comparison to those found in age-matched, or even younger subjects, which did receive a clinical diagnosis of AD [15–21]. These autopsy-confirmed findings greatly support the hypothesis that genetic or environmental factors might contribute to the clinical silencing of AD pathology, at least in a certain group of older subjects (e.g. the ASYMAD subjects). Moreover, these clinicopathologic discrepancies between brain pathology and clinical manifestations (of absence of them) in AD or generally in dementia, support, among others, the general concept of the “cognitive/brain reserve” [22–24].

The cognitive/brain reserve concept, though, needs to necessarily correspond to some biological reservoir of still-functioning cells and neural circuits, which ultimately result from the cumulative balance between beneficial and detrimental genetic and environmental factors that have impacted the architecture of the brain (e.g. synaptic contact distribution in the hippocampus) and that consequently have modified specific brain functions (e.g. memory) across all the previous periods of life [25]. Additionally, gene-environment interactions accounting for the cognitive/brain reserve capacities in a specific individual across his/her entire lifespan should consider possible prenatal genetic and behavioral factors originating from each parent since the fetal times [26–28].

*APOE* is one of the best examples that shows how a well-established genetic risk factor for an aging-related disease (AD) exerts its influence throughout the entire human lifespan and is part of complex metabolic and gene-environment interactions, especially in terms of brain cholesterol metabolism and synaptic formation [29]. The fact that the *APOE* polymorphism (presence of three possible alleles [*APOE2, APOE3, APOE4*] and for each individual the possibility to have only one of the six possible *APOE* genotypes [*APOE2/2, APOE2/3, APOE2/4, APOE3/3, APOE3/4, APOE4/4*]) correlates with different, sometimes divergent, clinical, pathologic, and survival outcomes, implies fundamental questions on which are the specific gene-environmental molecular mechanisms that determine those different effects across the entire human lifespan [30].

We aimed to briefly review some of the well-established findings on the *APOE* polymorphism associated with different, sometimes paradoxical, effects of this gene on the CNS structures and functions. We aimed to focus on human studies that evidence the effects of *APOE* polymorphism on the development of the brain, on different neuropsychiatric phenotypes, and on some higher cognitive functions present in humans only (e.g. language). Furthermore, we succinctly describe some of the possible molecular mechanistic aspects and biochemical pathways that have been proposed to explain the apparently paradoxical effects of *APOE* across the human lifespan [31].

## ApoE (protein)

In humans, the apolipoproteins E (ApoE2, ApoE3 and ApoE4) are molecules expressed in peripheral tissues (liver, spleen, kidneys, and macrophages) [32,33] and within the CNS [34]. ApoE is an essential apolipoprotein for the catabolism of triglyceride-rich lipoprotein constituents and it is found in chylomicron, low- and very low-density lipoproteins (LDLs, VLDLs) [35]. While liver and macrophages are the primary peripheral tissues where ApoE is produced, astrocytes are the main, and apparently the only cells within the CNS, that produce ApoE. Functionally, in the peripheral tissues, ApoE is part of cholesterol metabolism, while in the CNS it has been recognized as the principal carrier of cholesterol [36]. ApoE transfers cholesterol to neurons and represents an essential molecule for neuronal growth, synaptic plasticity, and membrane reparative processes [37]. The transfer of ApoE (cholesterol) to neurons occurs through the interaction of ApoE receptors [38]. These receptors such as LDLR, VLDLR, ApoER2, and LRP1 receptors belong to the low-density lipoprotein receptor gene family [39]. While the different chemical differences across the three isoforms of ApoE proteins started to be lately better defined [40], their different roles and effects in each cellular type remain to be completely understood yet [41,42].

## *APOE* (gene)

ApoE, the protein, is codified by a gene localized on the long arm (“q” arm) of the chromosome 19 in the sub-band 32 of region 13 (19q13.32): *APOE* gene (*APOE*). The *APOE* has three alleles (genetic polymorphism): *APOEpsilon*2 (*APOE*2), *APOEpsilon*3 (*APOE*3), and *APOEpsilon*4 (*APOE*4). Each allele codifies to corresponding protein isoforms (ApoE2, ApoE3, ApoE4) that differ in amino acid sequence at the residue 112 (also called “site *A*”) and 158 (also called “site *B*”). Specifically, *APOE*2, *APOE*3, *APOE*4 (the alleles) codify respectively for ApoE2, ApoE3, ApoE4 (the proteins), which at the position 112 and 158 of their amino acidic sequence, contain respectively cysteine/ cysteine, cysteine/arginine, and arginine/arginine [43]. These amino acid differences in the primary sequence of each ApoE protein determine a different number of charges (0, 1+, 2+) and account for their variant tertiary and quaternary conformations and electrophoretic differences [44]. Four individual mutations give electrophoretically separated bands at the E2 position. Using the isoelectric focusing techniques [45] at least four different bands (corresponding to different ApoE2 conformational status) have been identified: E2 (arg158-to-cys) [46], E2 (lys146-to-gln) [47], E2 (arg145-to-cys), and E2-Christchurch (arg136-to-ser) [48]. E2 (arg158-to-cys) is the most common of all four. In general, these “minimal” physical-chemical differences among ApoE proteins determine massive metabolic and structural consequences at tissue and metabolic level, especially within the human CNS [49,50].

## Genetic epidemiology

In the general population, the distribution of *APOE* alleles can vary across different ethnic groups [51]. These variations seem to be related to different non-genetic factors such as geographical latitude [52], local temperature and altitude, metabolic rate [53], hypoxia [54], “local” availability of lipophilic nutrients [55], climate [56] and others [57–60].

Notably, from an evolutionary point of view, *APOE*4 has been determined to be the “oldest allele” (which is also the only allele found in non-human primates and other mammals) followed by the *APOE*3, and then by the *APOE*2, which is indeed the “most recent allele” [61]. *APOE*2 has been estimated to appear in humans only about 80,000 years ago [62–64]. The more recent appearance of *APOE*2 in humans could be explained by the relative advantage provided by the corresponding product of this allele (*APOE*2 protein), which seems to increase the neural plasticity, synaptic reparative and clearance capacities of the CNS [65], as well as human longevity [66]. Intriguingly, however, while carrying the *APOE*2 could represent a protective factor late in life, especially for delaying dementia and cognitive deficits during aging, this is not necessarily the case early in life, especially during the very early phases of life (see antagonistic pleiotropy paragraph).

## *APOE* and Antagonistic Pleiotropy

**Figure 1:**
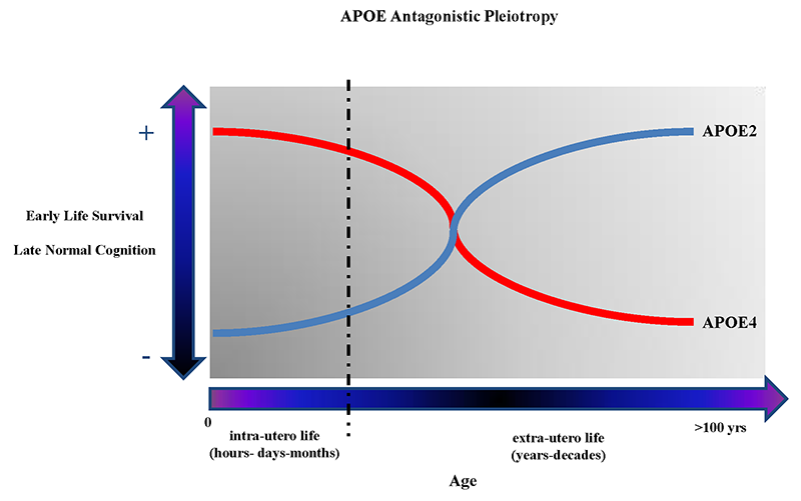
**The figure shows the theoretical antagonistic pleotropic (AP) effect of *APOE* gene across the entire human lifespan.** The increased probability of early survival and normal cognition later in life are alternatively correlated with the presence of *APOE4* and *APOE2* allele. However, other genes and environmental factors through their mutual interactions from the in-utero life until centenarian age can probably potentiate the beneficial or detrimental effects on the onset and manifestations of brain diseases and, respectively, reduce or potentiate the genetic predisposition toward more negative or positive clinical outcomes during different periods of life.

*APOE* began to appear as one of the best examples of genetic “antagonistic pleiotropy” (AP), a concept applicable to human traits and diseases, and to human cognition, neurodegenerative diseases, and brain diseases in general [67–71]. AP is defined as a genetic phenomenon existing when a gene can control for more than one trait (pleiotropy) with at least one possible trait beneficial and another detrimental (antagonistic) to the fitness of that same organism [72,73]. Once considered a rare genetic phenomenon, AP seems rather to be a much more frequent event, especially when considering human diseases [74], and in particular aging-related diseases and human longevity [75–77]. Among the *APOE* polymorphism-associated traits that appear to have AP characteristics are included the different biochemical consequences that ApoE2, ApoE3, ApoE4 proteins have on the metabolism of cholesterol, which have important implications, among others, on the metabolism of β-amyloid and synaptogenesis resistance in younger vs. aged mammalians, and could so explain the “paradoxical” beneficial effects of *APOE*4 on specific cognitive skills (e.g. verbal memory in schizophrenic patients [78], memory performance [79], and attention in young [80]), and the observation that *APOE*4 is an advantageous factor on survival and infertility in infectious environments [81,82], or that *APOE* alleles are associated with conscientiousness personality-trait in relationship to gray matter volume [7] and greater cortical connectivity [83]. Figure 1 shows graphs describing the antagonistic pleiotropy concept applied to *APOE* polymorphism.

## *APOE* polymorphism in normal and pathologic conditions of the brain across all ages

In general, *APOE*4 and its association with different measurable clinical variables such as dementia severity [84], neuropsychological scores [85], pathology burdens [86], cortical morphometry [5], morphometric-MRI [87,88], functional-MRI [89–91], electroencephalographic (EEG) [92,93] and magnetoencephalographic (MEG) signals [94], evoked potentials (EPs) changes [95,96], and other methods [97] has been investigated much more extensively than *APOE*2 [98–100]. In fact, studies focusing on *APOE*2 and its association to different clinical and subclinical parameters and possible molecular mechanisms of brain protection have been historically, less numerous than investigations comparing *APOE*4 vs. *APOE*3 for example. Furthermore, although *APOE*4 and *APOE*2 appear to have divergent effects on cognitive outcomes during aging, their corresponding protective or deleterious effects on other periods or conditions of life as brain development, birth prematurity, infancy, childhood cognitive and behavioral outcomes, as well as early-adult and mid-adult life predisposition for neurodegeneration have been generally much less investigated.

We organized the description of the reviewed findings on the effects of *APOE* polymorphism across the human lifespan using a bio-chronological order. We first described the effects of *APOE* polymorphism in normal and pathological conditions of the CNS during in-utero life followed by the neonatal, infancy, teenaging, and adult mid-life, and finally we describe data on *APOE* and the effect on elders and extreme old subjects including octogenarian, nonagenarian and centenarian populations.

### *APOE* and brain during in-utero life (before birth) and in preterm babies (<38 weeks of gestation)

Studies focusing on the possible clinical correlations between *APOE* polymorphism and divergent cognitive or behavioral outcomes of in-utero life, preterm babies, and infant populations are scarce. Due to its historical predominant consideration as a gene negatively associated with later life brain diseases, especially AD, *APOE* and its possible effects on the normal brain development or as a genetic modifier of specific pathologic conditions such as motor-behavioral delay, cerebral palsy, prematurity, and in-utero life brain abnormalities has received little attention until recent times [101]. In-utero life events imply complex interactions among maternal and fetal *APOE* genotypes, which might determine a highly complex spectrum of different biochemical interactions [102,103]. For example, recent findings propose that maternal *APOE* genotype could be a relevant risk factor for poor outcomes at birth, premature delivery, and predisposition for preeclampsia [27,104,105]. These maternal-fetal gene-metabolism interactions during in-utero life seem to potentially determine specific patterns of biochemical imprinting events directly at cerebral level, or alternatively, on different organs and systems such as the vascular system, which could indirectly include the cerebral vasculature [106,107]. However, based on more recent observations about AP phenomenon associated with *APOE* polymorphism and on more consolidated data showing that the *APOE*2 is, *per se,* a protective allele against dementia and cognitive decline during aging even independently on the β-amyloid accumulation and “classic” AD pathology [109], it would be possible in the future to investigate on unprecedented aspects of the cholesterol metabolism related to genetic aspects of *APOE* polymorphism whom influence seem to go well behind specific cognitive aspects and to be actually a “general modulator” of neuronal structures and functions, synaptic formation, brain repair, and neural regenerative mechanisms [110,111]. Consequentially, the *APOE* polymorphism appears to acquire an important general role in terms of brain development, impact on neuroreparative capacities and neuroplasticity potentialities across the entire human lifespan. Moreover, coherently with the concept of an early impact of *APOE* on brain structures, Stoknes et al. [112] have hypothesized that *APOE*2-carriers have a higher risk of death in-utero life following brain injury probably due to an altered or abnormal metabolism of the cholesterol. Other investigators have shown similar results and proposed similar *APOE*-based pathogenetic mechanisms [113]. These initial observations could explain why some findings show a non-significant association between *APOE* polymorphism and cerebral palsy (CP) simply due the fact that *APOE*2 carriage would represent actually a major risk factors for in-utero life survival (increased prenatal mortality). Recently, another group of investigators showed that *APOE* gene and weight at birth are risk factors for future cardiovascular diseases, and these factors seem to be independent factors as well [114]. To note, nonetheless, that these latter findings make the possible mechanistic (molecular) correlations among *APOE*, cholesterol metabolism, and vascular diseases much less linear than what could have been expected earlier [115,116]. Furthermore, data in support of the interaction between *APOE* alleles and environment suggest that a specific *APOE* genotype is a risk factor able to potentiate the toxic effects of various environmental contaminants. For instance, it has been shown that *APOE*4-carriage implies lower cognitive scores in preterm neonates exposed to higher levels of mercury [117,118].

### *APOE* and brain during normal neonatal (1 day-30 days full-term babies) and infancy (1-12 months full-term babies) periods

Studies on the impact of *APOE* polymorphism on various clinical outcomes in full-term normal neonates and infants are rare [119–121]. However, some investigations performed on selected types of neonate populations, suggest that *APOE* has indeed AP features. In fact, an over-representation of *APOE* 4/4 and 2/4 with a reduction in *APOE* 2/3 and 3/3 when compared with adult population [122] suggests that *APOE*4 is related to an increased risk of premature death. Furthermore, data from the same group of investigators found an over-representation of *APOE*2 in perinatal deaths populations [123]. This apparent paradoxical inversion of the protective effect of *APOE*2 vs. *APOE*4 in neonates, in contrast with the findings observed in elders, fits very well with the hypothesis that *APOE* has indeed antagonistic pleiotropic properties across the entire human lifespan [67,124,125]. Nevertheless, considering that the distributions of *APOE* alleles in healthy general populations are linked to various types of factors such as ancestry, geography, etc. (see paragraph Genetic epidemiology), much larger population cohorts stratified by the “local” *APOE* frequencies (corrected for all other identified influencing factors) together with a more precise definition of the periods of life to consider (perinatal deaths can have different definition across different countries) and large multi-institutional collaborative investigations are necessary to expand our knowledge on the possible pre-natal or peri-natal effects of *APOE* on early life events as well as on later life physiological or pathological events.

At a more speculative level, we retain that the possible early life effects associated with *APOE* polymorphism could primarily act on the cholesterol metabolism during the development of the brain and other organs and apparatuses (vascular system, liver, lungs, kidneys, etc.). Theoretically, *APOE* could also manifest a protective or detrimental effect on the brain development or its functions through indirect pathways [126,127]. Pivotal MRI-imaging studies focusing on morphometric aspects of gray matter (GM) in very preterm infants seem to predict some future aspects of neurodevelopmental outcomes [128]. While no imaging studies have been performed in large cohorts of normal full-term neonates or infants in the attempt to correlate GM morphometric aspects to *APOE* genotype, a link between *APOE* genotype and cortical thickness using neuroimaging techniques has been shown in young adults [129].

### *APOE* and brain during pathological neonatal (1 day-30 days) and infancy (1-12 months) periods

Another example of the possible negative effect of *APOE*2 on brain functions is the detrimental association between *APOE*2 and neurodevelopmental dysfunction observed in neonate and infants after cardiac surgery [130,131]. However, this potentially detrimental effect of *APOE*2 early in life could be biased by the higher incidence of neonatal sudden death associated with *APOE*4 or other genetic variants [132,133]. In fact, the supposed detrimental *APOE*2 effect in infants after cardiac surgery could be simply due to a possible skewing effect toward *APOE*2 (risk of a reduced survival) in the absence of the chance to test an equivalent number of *APOE*4-carrier subjects with similar conditions underwent to cardiac surgery and subsequently assessed. However, if this after cardiac surgery detrimental *APOE*2-cognitive outcomes association will be confirmed, it will corroborate the hypothesis that *APOE* has AP properties.

To date, the AP properties in humans are not well timed, particularly in relationship to the various phases of the brain development. It has been suggested that the AP effects of the genes related to the brain function could begin to be assessed only after the first year of life. This could be due to the fact, for example, that specific skills (e.g. motor skills) are “localized and matured” in specific circuits of the brain, only after certain periods of the brain development [134,135]. Consequently, it would be difficult, or impossible, at clinical level, to evaluate or pre-evaluate the possible future outcomes of each type of motor or non-motor skill until the full genetically-determined neurodevelopmental program has been completed. Furthermore, it is possible to hypothesize that not only there is a life-lasting AP effect of *APOE* across the entire human lifespan, but that this AP effect is present in different areas or brain circuits at different times of the development as well as in the same brain at different ages. In addition, it is not unconceivable to exclude that the AP effects might be additionally modulated by non-genetic factors (e.g. nutrition, environmental toxins, infections, etc.), especially before the conclusion of the major process of maturation of the CNS [136–138]. Of course, *APOE* is not the only gene involved in various highly complex phenomena of the human brain maturation but seems to be part of a cluster of genes that reciprocally interact during the different phases of the neurodevelopment [139]. Accordingly, it would be through these multiple short- and long-term interactions between parental genes and gestational environments that *APOE* polymorphism could influence neurodevelopmental trajectories during early life periods and infancy and later, by pre-establishing a higher risk for “aging-related” conditions, or at least influencing, clinical onset or phenotype in neurodegenerative diseases such as AD, Parkinson’s disease (PD) [140] or ALS [141]. Therefore, it is not possible to exclude that those specific metabolic abnormalities or environmental events occurring during the very early period of life could trigger specific biochemical compensatory mechanisms (including synaptic formation and biochemistry) in preterm babies, which are different from those possibly activated during a regular gestation period ending in full-term birth [142]. For example, there are data showing that children exposed to environmental intoxication can have a different cognitive impact based on the *APOE* genotype [143], and that these ApoE*-*toxicants interactions can be activated during earlier periods of life and establish the “neural ground” for potential future abnormal reactions in the context of a later normal environment. Finally, other types of illnesses such as intra-uterine infections could directly or indirectly and differently affect cognitive outcomes of children based on their specific *APOE* genotype [144].

### *APOE* and brain during normal childhood and teenaging

As investigations on the *APOE* polymorphism get performed considering human subjects with earlier age in life, the possible influence of each *APOE* genotype in these younger populations should consider that *APOE*2 and *APOE*4 can be part of AP phenomena occurring in the same individual (e.g. *APOE*2/E4) and across different individuals (e.g. *APOE*3/E2 vs. *APOE*3/E4) at different ages. The AP effect of *APOE* suggests that it would be fundamental to precisely define the specific periods of life to investigate and in which of those periods AP effects take place [145]. In fact, during earlier periods of life, the deleterious effects of *APOE*4 vs. *APOE*2 appear to be paradoxically reversed in some cases in respect to older age [31,129,146,147]. Although studies focusing on the allelic effects of *APOE* on young adults and children are relatively rare, a series of recent analyses evidenced an increased risk for poorer outcomes in *APOE*2- vs. *APOE*4-carriers in pediatric populations [130]. However, this effect is paradoxical only in appearance if the AP concept is considered. The “switched” effect of *APOE*4 (and alternatively of *APOE*2) during aging (in comparison to earlier periods of life), fits very well with the genetic AP concept for which the function of a gene can change during the time (i.e. from infancy to extreme age) or it can be otherwise activated in presence of different or mutated environmental conditions [148]. In support of the AP properties of *APOE* alleles, recent imaging studies have demonstrated a negative correlation between *APOE*4 and GM maturation in subjects between 3 and 20 years old [149]. However, more specific aspects of cortical connectivity and cognition - such as fluid intelligence - resulted to be lower in *APOE*4-carriers vs. non-*APOE*4-carriers [150]. Yet, in this last study, no direct analyses were performed to compare *APOE*4- vs. *APOE*2-carriers. Other studies, however, have also analyzed the interaction between *APOE* (e.g. non-*APOE*4 carriers) and other genes such as SORL1 showing that is the interaction between these two genes to determine different levels of hippocampal connectivity [151]. Again, when studying the *APOE* effects across different ages, it looks like of extreme relevance to define in a very precise way, the quantitative parameters or qualitative variables (including other genetic traits or genes) to analyze (e.g. clinical signs or symptoms, cognitive scores, imaging measures, neurophysiological values, etc.) and the specific system, organ, tissue or function to study.

### *APOE* and brain during pathological conditions of childhood and teenaging

There have been various studies that focused on neurological disorders during childhood and adolescence that attempted to identify possible meaningful correlations between manifestation and progression of a disease, or its variable phenotypic characterization, and the *APOE* genotype. Initial findings by Treble-Barna et al. [152] suggest an *APOE*-environmental interaction in terms of long-term outcomes in children with a history of traumatic brain injury (TBI). This possible gene-environmental interaction has been further suggested by Kassam et al. [153] based on meta-analytic results showing that *APOE*4 is undoubtedly associated with a worse prognosis after a TBI in both children and young adults and that this effect might be due to mechanisms quite different from those linked to neurodegeneration during aging.

In support of the hypothesis that *APOE*4-related effects could be linked to different pathomechanisms than the ones leading to neurodegeneration when considering short- or long-term outcomes in the context of the same disease, for example traumatic brain injury (TBI) where there are data showing the possible association between *APOE* and post-concussive syndrome in children, which do not have any significant difference between *APOE*4- vs. non-*APOE*4 carriers, in particular when immediate or short-term outcomes are considered [154]. By contrast, in other types of brain diseases such as epilepsy, *APOE* genotype resulted to be a strong modifying factor in terms of both neuronal and glial neuropathological changes [155]. Nonetheless, depending on the specific type of disease associated with seizures or epileptic syndromes, the impact of *APOE* polymorphism seems to vary and it is not always associated with a direct epilepsy-inducing pathogenetic effect [156], but rather with other clinical aspects such as the age of onset of the disease [157].

In summary, *APOE* polymorphism during childhood and teenaging does not seem to be necessarily associated with specific structural brain changes but does seem to act in a much more complex way on a series of clinical outcomes characterizing the natural history of a specific disease such as cognitive decline, age of onset, or other associated co-morbidities related to the primary pathologic condition [113]. Though, earlier in life, the influence of *APOE* genotype seems to operate on a wider set of aspects either at the structural as functional level [158,159].

### *APOE* and brain during normal adulthood (3^rd^-7^th^ decade of life)

A series of *APOE*-associated white matter (WM) and gray matter (GM) microstructure differences in adult life have been described and they support the hypothesis that *APOE* polymorphism has indeed an early impact on various neurocytological aspects and different neurophenotypic aspects during normal and pathologic conditions of the CNS. For example, recent cognitive-morphological MRI investigations in normal adult populations described an earlier influence of the *APOE* genotype on specific morphometric aspects of different brain regions as an independent variable that is, as a variable that is not linked to any other known associated genetic or environmental risk factor [83,160–164]. Moreover, functional MRI (fMRI) studies showed significant differences in normal older as well young-adult populations when clustered based on *APOE*4 vs. non-*APOE*4 carriers [165–168]. In addition, other factors such as brain metabolism [169] and vascular reactivity [170] seem to be associated with an *APOE* genotype, which predisposes to cognitive impairment later in life.

Although studies using normal human brain tissue for comparisons across different *APOE* genotypes are very rare, Love et al. [171] were able to demonstrate different levels of synaptic proteins in the temporal cortex of normal brains between *APOE*4 vs. non-*APOE*4 carrier subjects.

### *APOE* and brain during pathological conditions in adulthood (3rd-7th decade of life)

There are different investigations demonstrating that *APOE* genotype can influence the clinical phenotype of brain diseases during adulthood. For example, different levels of neuroinflammation associated with epilepsy were directly correlated with a specific *APOE* genotype in adult patients [172]. Specifically, *APOE*3 conferred a higher level of protection against neuroinflammatory and neurodegenerative processes associated with epilepsy [155]. In other brain diseases, such as TBI, it has been shown that different clinical outcomes are present in young adults (in contrast with findings in children) *APOE*4-carriers vs. non-*APOE*4 carriers [173,174]. In this case, it has been shown that a specific *APOE* allele can directly influence the reparative capacities of the brain and consequently the clinical outcomes based on a specific allele, the *APOE*4 in this case [175].

Another fascinating set of data proposed that there is a significant association between sleep disorders and *APOE* genotype [176,177], as well as among *APOE*, sleep-wake cycle, and deposition of *β*-amyloid [178]. This is a further indication that during adult age *APOE*4 confers negative effects to those subjects that have sleep-disordered breathing illnesses. By contrast, it has been evidenced that *APOE*2 confers a higher risk for intracranial hemorrhage in the contest of the natural history of brain arteriovenous malformations in young adults [179].

Then, importantly, as the mean age of the subjects analyzed in each different clinical or epidemiological study becomes lower, it is valuable to consider the possible AP effect of *APOE* alleles together with a clear distinction of the pathologic conditions to investigate, for example a clear distinction should be kept if a specific study is considering a vascular vs. non-vascular diseases. In fact, as the mean age of the subjects analyzed across different studies lowers also the different type of tissues, organs, or functional systems to target could vary together with the notion that an AP phenomenon can affect different organs or functions at different biological periods of life as well as different areas of the same organ (e.g. the brain) across those different periods of life.

### *APOE* and brain during normal aging: successful octogenarians, nonagenarians, and centenarians

To date, *APOE*4 remains the best-established genetic risk factor associated with an increased risk of sporadic late-onset Alzheimer’s disease (LOAD) [180,181]. The possibility of *APOE*4 to determine an increased risk for other types of dementias is currently debated and under continued investigation [182–186]. While *APOE*4 has a deleterious effect on the onset and progression of AD as well as on cognition during pathological aging [187], *APOE*2 - the rarest allele of *APOE* gene in Caucasian populations - has been associated with a reduced risk of AD and increased longevity [77,188,189]. However, the detrimental effect of *APOE*4 on cognition (in both homo- and heterozygosis) has been largely confirmed by clinical [190], neuropsychological [191], neuroimaging [192], and neuropathological studies [193]. Conversely, fewer investigations have focused on the impact of *APOE* in normal brain aging conditions and specifically on *APOE*-related influence on cognition and other brain functions during normal brain aging. Furthermore, for statistical purposes, *APOE*4 effects have been much more frequently contrasted to *APOE*3 (the most frequent *APOE* allele in Caucasian populations) and much less to *APOE*2 due to its lower frequency in most human populations [194]. Very often, in fact, the rarity of *APOE*2 did not allow performing a correct and reliable statistical approach due to the marked differences in terms of population sample sizes to compare (e.g. numerosity of *APOE*4 vs. *APOE*2 subjects).

Moreover, the harmful effect of *APOE*4 has been associated not only to a higher risk of AD but also to a higher risk or different clinical outcomes for other neurological [195–198] and non-neurological conditions [199–203]. These latter findings support the hypothesis that *APOE* is very much indeed a possible general modifier of various biological and consequently, clinical phenomena. Curiously, *APOE*4 has also been associated with some apparently paradoxical effects [204–207]. For example, the absence of a higher risk for AD or cognitive deficits in some specific human populations has been found [206,207]. Nonetheless, the variability of *APOE*4-associated risk across different human populations not only reinforces the concept that *APOE*4 is “just” a risk factor (in contrast to a Mendelian mutation) but also that other environmental and genetic factors are possibly associated with a higher risk of dementia, its onset, and pathogenic progression [208,209]. Significantly, the detrimental or beneficial biological mechanisms of *APOE* alleles during premorbid normal adult life periods are far from being completely clarified.

In terms of *APOE* polymorphism-associated changes during normal aging, some studies have shown that *APOE*4 (vs. *APOE*3) is associated with a cortical thinning in a series of specific brain regions (e.g. medial and inferior temporal regions, including entorhinal cortex) [210]. These thinner cortical regions are the same cerebral regions more frequently affected in AD and particularly vulnerable to the AD pathology accumulation (e.g. β-amyloid accumulation). Interestingly, though, the *APOE*4-associated thinning of those AD-vulnerable cortical regions seems to be, per se, independent from the extracellular β*-*amyloid accumulation, especially when cognitively normal vs. early mild cognitive impairment (EMCI) or AD subjects are taken in account [210]. This newer perspective on *APOE*4 that is, a genetic factor having its own impact independently from β-amyloid accumulation (or other pathologies?) seems to confirm a direct and autonomous influence of *APOE*4 on the cortical architecture in specific regions of the brain [211,212].

The pathogenetic “independency” of *APOE* could be probably linked to basic cholesterol metabolic mechanisms related of the three different *APOE* alleles, their final protein products and cellular localizations [213]. In addition, the possible association among *APOE* genotypes, specific brain areas architecture and related brain functions, seems to go behind pure cognitive aspects and involve other non-cognitive functions such as the motor skills for example [214].

Finally, it is relevant to consider that the effect of *APOE*4 on the pathology and progression of AD, and probably on human cognition in general, appears to be mitigated by a series of other possible beneficial environmental factors [215]. However, it is not completely known which could be the possible environmental factors that could alternatively (beneficial or detrimental) potentiate the opposite effects of *APOE*4 and *APOE*2 during normal life conditions.

This brief description of some most recent neuroimaging and population-based studies of the *APOE* impact in humans illustrates either the complexity and intrinsic plastic capacities of the human brain to produce different clinical outcomes as consequence of the mutual interplay among genetic risk factors (e.g. *APOE*4, is not a determinant of disease) on the neural tissue (e.g. cortical thinning), independent pathogenetic factors (different for example, from β-amyloid extracellular accumulation or intracellular phosphorylated-tau formation) and other possible environmental variables, which are not identified or completely investigated yet such as lifestyle, nutritional habits, stress, or history of infectious diseases. Moreover, it is important to mention that there are recent findings from relatively large cohort studies that enrolled cognitively normal centenarians, which started to investigate cognitive aspects related to *APOE* genotype at a very advanced age and started to show unexpected and interesting results. For example, *APOE*4 in centenarians seems to be positively correlated with negative rather than positive affect in interaction with life events [216].

In general, these new findings seem to reinforce the concept that *APOE* not only influences some cognitive aspects until very late in life, until the 10^th^ of life or later, but that there are much tighter possible links among cognition, emotions, and longevity in the human species [217].

### *APOE* during pathological aging: Alzheimer’s Disease and other dementias in elders

In the context of neurodegenerative diseases, and dementia in particular, extensive analyses have shown that *APOE* genotype influences cognitive and non-cognitive phenotypes of subjects with a clinical diagnosis of MCI or AD (clinically probable AD). For example, *APOE*4 has been found to cluster at least two different groups of cognitive outcomes among subjects with dementia [5]. Moreover, in behavioral terms, *APOE*4 has been associated with different compartmental subtypes of AD [218]. Other investigations have further demonstrated that *APOE*4 can generate different endophenotypes in terms of neuropathology. For example, Murray et al. [219] were able to distinguish different subtypes of AD when *APOE*4 was clustered together with age of onset of the disease. However, in other non-AD dementias it seems that the detrimental link between *APOE*4 and cognitive decline is much more complex than in AD [220,221]. Moreover, previous and more recent investigations began showing that in the context of non-AD dementias such as FTD or cognitive decline in ALS, the modifying action of *APOE* polymorphism can be rather different from what is observed in classical AD [222,223]. For example, cognitive dysfunctions in ALS are associated with *APOE* genotype and other genes as well, such as SCNA gene. In this case, it seems that both genes cooperate for a higher risk of dementia in ALS only when related to the presence of Lewy bodies - a typical neuropathological feature of dementia with Lewy bodies (DLB) [224,225]. These recent findings support the possible mutual interactions between *APOE* and other genes and other non-genetic factors and that these more complex molecular interactions can produce different clinical phenotypes during the manifestations of age-related neurodegenerative diseases and probably earlier in life as well [226,227].

## The “special” case of *APOE*2 and neurodegeneration during aging

As described earlier in this review, only few studies have focused on the relative impact, including possible molecular mechanisms, of *APOE*4 vs. *APOE*2 in terms of different cognitive outcomes during normal or cognitively successful aging [228–231]. Those few studies that have attempted to verify the possible associations between cognitively normal older subjects and *APOE* polymorphism have shown that the protective effect of *APOE*2 is not directly associated with mechanisms linked, for example, to the extracellular β-amyloid accumulation into the brain or to the alterations of synaptic and neuroinflammatory levels in the CNS [232,233]. These latter observations are of special interest since they could open other and different views on the possible protective mechanisms of *APOE*2 independent on the “classic” AD pathology [234,235]. These more recent findings allow hypothesizing that the protective effects of *APOE*2 on the brain and its functions are intrinsic to the molecular features of *APOE* alleles and its protein products. In fact, the beneficial effects of *APOE*2 later in life can be only partially, or indirectly, related to the reduction of those pathologic factors currently considered as the “pathogenetic” causes of AD: extracellular accumulation of β-amyloid pathology, hyperphosphorylated-tau formation and spreading, increased levels of neuroinflammation and synaptic loss [236,237]. Therefore, these more recent findings imply that also other factors linked to the intrinsic molecular advantage of *APOE*2 need to be taken in account [228,238,239]. However, the new molecular aspects of *APOE*2 need future confirmation by analyzing data from very large prospective studies aiming to measure the real effect of *APOE*2 protein in a large multi-factorial statistical model that would include both biological (genetic) and non-biological (environmental) factors [240,241]. These studies should include human brain donations to increase the chance to quantifying ratios between residual (normal/still-functional) and pathologic tissues (dysfunctional neuronal or non-neuronal) that could directly derive from the action of *APOE*2 vs. *APOE*4 on the neural tissue through probably its biochemical interaction with cholesterol metabolism. Therefore, it could be possible to hypothesize that the *APOE*2-related action on the cholesterol metabolism would be capable to delay the cognitive decline during normal aging by specific molecular mechanisms that could be modulated using pharmacological compounds or non-pharmacological treatments, or alternatively, offer the opportunity to potentiate their beneficial action in a more preventive or protective manner. Unfortunately, the specific *APOE*2 molecular mechanisms are currently not completely known [242]. Nonetheless, some molecular mechanisms have been proposed based on new and unexpected metabolic links between different biochemical pathways. For example, new possible links between homeostatic pathways of iron and lipids metabolism [243] or between *APOE*2 and disease-onset of other mutations through the interaction of *APOE*2 and genes involved in cellular proliferation, protein degradation, apoptotic and immune dysregulation processes has been shown [244]. Moreover, differential phenomena of neuroplasticity as based on *APOE*4 vs. *APOE*3 have been proposed [245]. Furthermore, as for possible differences across the three *APOE* proteins, recent pilot investigations started to show that *APOE* genotypes is associated with different plasmatic levels of *APOE*2 vs. *APOE*3 and *APOE*4 proteins as hourly measured in human subjects [246]. These newer biochemical findings on *APOE* have been made possible by recent methodological advancement especially those based on mass spectrometry [247] and highly complex techniques [248].

## *APOE*, higher cognitive functions, and modulatory effects of personality-traits

*APOE*2 in older subjects, even in the presence of high levels AD pathology and clinically silent dementia, have shown a significant association with higher language skills acquired early in life [249]. Furthermore, recently, MRI findings showed that specific spectroscopy signals are associated with cognitive and language development at term-equivalent period in some of the identical brain areas (e.g. hippocampus) that are later often affected by aging-related diseases such as AD [250,251]. However, it is not known, yet, if these imaging findings are also directly linked to *APOE* or to other genetic polymorphisms. Nonetheless, it is highly possible that language skills, as well as other higher cognitive human functions, could be connected to more specific gene-environmental interactions during either in-utero life and during the very first months or years of life as based on a cluster of genes, including *APOE*, whose function or dysfunction, directly predispose an individual to a higher risk of neurological and psychiatric risk later in life due to the very early impact of those gene-environmental interactions on specific brain structures and functions “already set up for” at birth or earlier. These early gene-environmental neurobiological phenomena could predispose to specific types of neurophenotypes later in life during the youth, middle-age or aging of that individual [253].

Intriguingly, *APOE* genotype seems to be associated or interacting with specific traits of personality [254], and even with GM volumes in specific areas of the brain that can have a modulatory effect on cognition. Among the personality traits that seem to be associated with an *APOE*-personality modulatory interaction there is the conscientiousness [7]. Although many of these studies were limited by the relatively small number of subjects analyzed, they do represent initial and important findings of the more plastic relationship between *APOE* and personality-based behavioral aspects that could represent useful clinical tools in establishing a higher risk of dementia before any clinical manifestation [255,256].

## FUTURE PERSPECTIVES

This necessarily succinct review focusing on the possible types of impact of *APOE* polymorphism on human brain structures, cerebral functions, and brain illnesses across different ages in normal and pathological conditions of the brain and possible modulatory effects of specific personality traits (e.g. conscientiousness, neuroticism) suggests that future larger longitudinal investigations would need to consider numerous genetic and environmental variables to fully identify the molecular mechanisms of *APOE* as well as the interactions between the *APOE* with other genes and other aspects such as environmental imprinting (nutritional, educational, behavioral, etc.) during the early phase of life [257,258].

*APOE* started to appear a gene associated with relevant antagonistic pleiotropic phenomena and it seems to be involved in different human diseases, not only brain diseases [259]. *APOE* appears, in fact, to have an important and modifying impact on lung diseases [200], infectious diseases [202,260], and on very specific pathologic processes [203]. Finally, *APOE* seems to be involved in the different behavioral outcomes and personality-traits modulatory effects whose molecular mechanisms still need to be completely elucidated [261].

Future studies, possibly stratified by individual *APOE* genotype, should be able to identify the precise molecular mechanisms by which a specific compound could modify or halt the detrimental effects of *APOE*4 during late-life period or, by “paradoxical” contrast, enhance its effects during the early or very early phases of life. Different types of molecules with possible pharmacological actions on the *APOE* gene products, and especially on *APOE*4 gene product, are under constant investigation [262,263], using newer technological sequencing and computational advancements that better inform us on the possible genome-based mechanisms of health maintenance [264,265]. Moreover, *APOE*4 has been identified as a ligand for the LDL receptors (LDLRs) family and that ApoE receptor 2 (ApoEr2) [266], together with VLDL receptor (VLDLR), have major impacts on brain development and adult synaptic plasticity [267,268]. ApoEr2 acts through the activation of Reelin [269–271], whose function could be modulated by specific compounds that secondarily would act or reduce the *APOE*4 detrimental effects on the CNS in general, or on dementia being the main genetic risk factor for AD [272,273].

Finally, new and exciting facets of CNS biology and aging such as those related to the glymphatic system [274], or the CNS-associated lymphatic system [275], have recently opened new ways to better understand the CNS biology, its clearance capacities, as well as the possibility to associate these new discoveries with acquired knowledge about *APOE*, its pathogenic contribution to neuropathology of dementia, and the effects of *APOE* alleles across the entire human lifespan.

## BOX 1. POSSIBLE *APOE*-LINKED BIOLOGICAL TRAITS ASSOCIATED WITH ANTAGONISTIC PLEIOTROPY FEATURES

Although this review aimed to describe the findings about different functional brain outcomes, in normal and pathological conditions of the brain, associated or influenced by the *APOE* allelic polymorphism in humans, we also briefly describe some of the possible *APOE*-molecular and non-molecular traits that could be associated with the proposed AP features of *APOE*. There is a plethora of animal studies (experiments employing transgenic animals, mainly rodents) that define various genetic (*APOE*), metabolic (ApoE proteins), and ApoE receptors (intracellular functional consequences) molecular aspects that would be impossible to describe here in an exhaustive way. For this reason, we preferred to focus on those possible *APOE*-traits that seem to have some antagonistic pleiotropic (AP) features across the lifespan in mammalians.

We retain that the following “*APOE-*linked traits” are among the most suitable candidates able to explain the antagonistic pleiotropic effects of the *APOE* polymorphism:

### *APOE4*-carrier as “Higher β-amyloid accumulator subject”

Using different animal models and different biochemical approaches, it has been demonstrated that *APOE*4-animals have a reduced capacity to catabolize and consequently to reduce, the progressive accumulation of 1-42 β-amyloid, one of the main constituents of extracellular β-amyloid accumulation [276–278]. Furthermore, *APOE*4 seems also to indirectly exacerbate tau-mediated neurodegeneration [279]. Moreover, the *APOE*4 seems to determine this reduction of catabolic 1-42 β-amyloid (as well as of other forms of β-amyloid [280,281]) in a regionally-based manner [282–284]. If these deleterious effects of *APOE*4 on the β-amyloid metabolism during aging or adult life are deleterious as well for the brain or other tissues in early life is not known or, by contrast, if *APOE*2 has a beneficial effect in early-life is not completely understood [285,286]. Curiously, however, newer investigations both in animals and humans showed a “paradoxical” (due to actually to the AP?) effect of *APOE*2, specifically in case of *APOE*2 homozygosis (*APOE*2/E2) [287]. Zhao and colleagues, for example, show increased levels of tau lesions in cases of Progressive Supranuclear Palsy (PSP), a typical tauopathy, when associated with *APOE*2 homozygosis.

### *APOE4*-carrier as “Lower synaptic/spine replacement/ repairing capacity subject”

A consistent series of experiments either in-vitro and in-vivo aiming to compare the formation of synapses or the capacity to repair synapses in the presence of *APOE4* vs. *APOE*3 or *APOE*2 have now consolidated the evidence that *APOE* polymorphism is indeed directly linked to differential capacities of the brain, both in terms of synapses formation and repair, when one of those three alleles are expressed. For example, in the specific context of AD - where entorhinal cortex lesions represent some of the very initial pathogenetic events ending in the synaptic loss subjacent memory impairment - it has been demonstrated that *APOE*4 is associated to a reduced capacity to form new synapses as measured by GAP-43 and synaptophysin proteins, which are typical molecular markers of neo-synaptogenesis [288,289]. Furthermore, other investigators have demonstrated that *APOE*4 is specifically associated with a reduced capacity to produce spine and consequently contribute to the dendritic complexity [1,290]. Although these animal models show the detrimental effect of *APOE*4 on the synaptic compartment, which is coherent with the *APOE*4 effect later in life, paradoxically, or better in antagonistic pleiotropic terms, there are studies in humans showing a higher performance of specific cognitive tasks in younger subjects when carrying *APOE*4 in comparison to *APOE*2 [291–293]. However, some new recent studies in humans show that even in the presence of *APOE*4 there are some morphometric alterations of the dendritic spines (e.g. in the frontal cortex), which could support the maintenance of normal cognitive functions despite the presence of AD pathology or aging [294].

### *APOE4*-carrier and personality-traits, “Higher genetic-personality-trait susceptible subject”

The neurohistologic structure as the neuronal and synaptic complexity associated to the different traits of personality defined in humans is not completely known [295]. The attempt to link specific connectomics aspects to each specific type of personality or behavior is still in its infancy [296–298]. However, initial studies are showing interesting findings when introducing the *APOE* polymorphism factor in the analyses of possible connectomics changes in *APOE*4 carrier individuals. Some recent studies [300,301] show that the default-mode network in *APOE*4 subjects differs from non-*APOE*4 in a way that *APOE*4 seems to establish the functional communications across different brain regions (connectomics), which could predispose to some cognitive disadvantage across the entire lifespan. Moreover, some investigations pointed out that there is indeed an association between cognition measurements and personality traits [302,303]. If these links are also based, influenced, or modulated by a specific genotype, such *APOE*4 vs. *APOE*2 is under investigation. However, initial studies, show that specific personality factors (e.g. neuroticism and extraversion) can actually moderate the cognitive outcomes in *APOE*4 carriers [255,261].
